# Challenges and opportunities: a mixed-methods study on the current status of head and neck cancer support groups in Australia

**DOI:** 10.1007/s00520-025-09752-8

**Published:** 2025-07-11

**Authors:** Xiaojing Zhou, Ashleigh R. Sharman, Rebecca L. Venchiarutti

**Affiliations:** 1 Sydney School of Public Health, Faculty of Medicine and Health, The University of Sydney, Camperdown, NSW 2050 Australia; 2 Department of Head and Neck Surgery, Chris O’Brien Lifehouse, 119–143 Missenden Road, Camperdown, NSW 2050 Australia

**Keywords:** Head and neck cancer, Head and neck neoplasms, Support groups, Health services, Mixed methods

## Abstract

**Purpose:**

Head and neck cancer (HNC) support groups provide valuable emotional support and practical guidance, helping to improve patients’ quality of life. This study aimed to understand the current practices of HNC support groups in Australia, focusing on their establishment, implementation, and facilitation, and identifying gaps in resources, guidelines, and training for facilitators.

**Methods:**

This study recruited facilitators of active HNC support groups in Australia. A mixed-methods approach was used, combining quantitative and qualitative analyses. Quantitative data were collected through an online questionnaire and descriptively analyzed. The qualitative data were analyzed using inductive content analysis of open-ended questionnaire responses.

**Results:**

Nineteen participants completed the survey. One-third of HNC support groups are in rural areas, two-thirds in urban areas. Nearly half of the groups operate in hospitals, with only one online support group in remote areas. Key challenges identified were administration, content, and location. About 32% of participants received guidelines, and 42% received training, with most finding these resources useful. The need for more online support groups and collaboration with professional bodies was highlighted.

**Conclusions:**

HNC support groups face significant administrative and logistical challenges. Support groups in rural areas also encounter greater access issues, making online formats potentially more sustainable. Future efforts should focus on improving administrative support, funding, and comprehensive training for facilitators to ensure the long-term sustainability of HNC support groups in Australia.

**Supplementary Information:**

The online version contains supplementary material available at 10.1007/s00520-025-09752-8.

## Introduction

Head and neck cancer (HNC) comprises a group of malignancies occurring in the head and neck region, including the oral cavity, larynx, pharynx, nasal cavity, and nasopharynx, and is the seventh most common cancer worldwide [1]. According to the Australian Institute of Health and Welfare, the age standardized incidence rate of HNC in 2021 was 16.7 cases per 100,000 people [2]. People with HNC may experience long-term sequelae resulting from cancer and its treatment, while also facing various challenges associated with surgery, radiotherapy, and chemotherapy [3, 4]. Based on the location of the cancer, people with HNC may suffer physically from pain, changes in voice, limited ability to eat and swallow, and they may also experience more noticeable changes in the structure of their face and neck [5]. The psychological burden among individuals with HNC may be exacerbated by pain, visible disfigurement, and functional impairments (e.g., speech or swallowing difficulties), increasing their vulnerability to anxiety and depression [6]. Therefore, survivors of HNC may face complex ongoing needs, including managing the long-term effects of cancer treatment and significant physical and psychological rehabilitation demands [7, 8].

Cancer support groups offer a wide array of resources to their members, including a sense of community and security [9, 10]. These groups create a unique environment where people can connect with others facing similar challenges, thus providing them with invaluable emotional support and practical guidance [9, 11]. While several studies have acknowledged risks that participation may lead to emotional distress, especially when individuals are confronted with other participants with advanced illness, have difficulty relating to others, or are exposed to emotionally triggering discussions [12–14], participation in HNC support groups may also contribute to improved quality of life following treatment [15, 16]. Compared to non-participants, people with HNC who engage in support groups demonstrate significantly better outcomes in areas such as diet, emotional well-being, pain management, and overall distress, as well as in assessments of HNC cancer-specific quality of life [16].

In Australia, the majority of cancer support groups are non-therapeutic peer support groups, either professional or peer-led social support groups, or “self-help” groups typically facilitated by individuals with personal experiences of cancer [17]. Although support groups play a key role in improving patient well-being, they are often ad hoc and without a systematic approach to implementation and up-to-date practice guidelines [18]. Furthermore, existing research predominantly focuses on the experiences and significant challenges faced by HNC patients in terms of their physical and emotional quality of life post-treatment, as well as their needs for support and information [19–21]. However, there is a lack of research that examines whether HNC support groups can effectively meet the multifaceted needs of these patients, how these groups can operate more efficiently, and how they can achieve sustainable development.

This study aims to better understand the current practices of HNC support groups in Australia. The objectives are to describe how these support groups are established, implemented, and facilitated, and to identify gaps in the resources, guidelines, and training they provide for facilitators. The ultimate goal is to make recommendations to improve sustainability of services offered by existing and newly formed HNC support groups.

## Materials and methods

### Study design

A mixed-methods study design was used to assess the characteristics, gaps, and sustainability of HNC support groups in Australia, including descriptive analysis of questionnaire responses and qualitative content analysis of free-text questionnaire responses [22]. Ethics approval was granted by the Sydney Local Health District Human Research Ethics Committee (HREC) (Protocol No. X23-0462 & 2023/ETH02485). Research governance approval was granted by the Chris O’Brien Lifehouse Research Governance Office (LH23.072).

### Participants and recruitment

This study focused on active HNC support groups in Australia and aimed to recruit a minimum of 20 HNC support group facilitators as participants. This target was based on a previously identified pool of 26 active HNC support groups in Australia [23], with the intention of achieving a high response rate (around 80%) while ensuring diversity in geographic location, group structure, and facilitation type. Participants were eligible if they were a facilitator of existing HNC support group within Australia, including patient advocates, allied health staff, nurses, and other clinical staff. Individuals who facilitated support groups that were not specific to HNC were excluded. Participants were identified from the consumer advocacy organization Head and Neck Cancer Australia (HANCA)’s website listing patient support groups [24], and this list was cross-checked against the 2023 national review [23]. Eligible participants were sent a link to the online survey by a member of the study team. The study was also advertised using free options through the social media channels (X [formerly Twitter], LinkedIn, Facebook) of associated organizations including Chris O’Brien Lifehouse, HANCA, and individual investigators.

### Questionnaire design

The questionnaire was divided into three sections: participant demographic information, HNC support group foundational information, and support group training information.

Section 1 collected demographic and professional information about participants using primarily closed-ended questions, such as multiple choice for gender, age, and employment status.

Section 2 collected information about the characteristics and operations of the support groups facilitated by the participants. It included both closed-ended questions (e.g., multiple choice for the location and setting of the support group) and open-ended questions for additional details like the year the support group was established and other specific characteristics not covered by predefined options.

Section 3 assessed the training and guidelines available to participants, with a mix of closed-ended questions regarding the existence and utility of guidelines and training, and open-ended questions allowing for detailed explanations of the effectiveness of these resources and the specific challenges faced in managing a support group.

Our study’s questionnaire underwent a pilot testing phase prior to dissemination to ensure its effectiveness and clarity. This preliminary testing was conducted internally among the study team members to refine the survey instrument and to preemptively address potential issues.

### Data collection

Prior to completing the survey, all participants were provided with a Participant Information Sheet (PIS). Informed consent was implied by completing and submitting the anonymous online survey hosted on REDCap [25]. Data were collected from January 2024 to March 2024 and all data were collected in the REDCap database.

### Analysis

#### Quantitative analysis

Quantitative data were descriptively analyzed using jamovi (Version 2.3.28.0) and Microsoft Excel, and are presented using frequencies and percentages. No inferential statistics were undertaken. Additionally, to quantify the impact of each challenge in Section 3, a scoring system was used in which the issue deemed most challenging by a participant was allocated 3 points, the second most significant received 2 points, and the third most significant was assigned 1 point. The scores were summed for each item and items ranked based on total scores, with a higher score indicating the item was perceived as being a greater challenge for support groups.

#### Qualitative analysis

Qualitative analysis of the data consisted of an inductive content analysis of the participants’ open-ended responses about their experiences of the support group and the training they received for the support group [22]. One member of the research team (XZ) conducted multiple readings and open coding of the open-ended questionnaire responses, summarizing the main category for each open-ended question as individual units. Following the preliminary organization of the qualitative data, the research team (RV, AS, XZ) held several meetings to review and refine the highlighted text from the original data, ensuring the accuracy and consistency of the generic categories. Finally, the abstraction process involved synthesizing these main categories to develop a comprehensive understanding and description of the operation and training within the HNC support groups.

## Results

### Demographic characteristics

Participant characteristics are shown in Table 1. Sixteen participants completed the entire questionnaire, and three participants completed a sufficient proportion of the questionnaire to be considered useful for analysis and were also included. Therefore, a total of 19 participants were included in the analysis. Of the 19 valid questionnaires, 12 facilitators (63%) were healthcare professionals, and nine (48%) were cancer survivors or patients. Accordingly, the majority of groups were facilitated either by healthcare professionals or individuals with lived experience.

**Table 1:** Participant characteristics

Characteristics	Participants *n* (%)
Gender	
Male	7 (37)
Female	12 (63)
Age (years)	
18–29	1 (5)
30–45	4 (21)
46–59	7 (37)
60–75	7 (37)
Background	
Clinical	12 (63)
Non-clinical	7 (37)
Employment status	
Full-time	9 (48)
Part-time	4 (21)
Freelance	1 (5)
Retired	5 (26)
Previously diagnosed with HNC	9 (48)
Previously cared for people with HNC	4 (21)
Holding relevant qualifications in support group facilitation	
Yes	5 (26)
No	9 (48)
Not sure	5 (26)

### Support group characteristics

The majority of support groups were established between 2000 and 2020 (63%) (Table 2). The three groups established before 2000 had been established as far back as 1958, 1973, and the 1990s. Most support groups were in urban areas or major cities (68%). Almost half of groups operated within public hospitals (48%), followed by community spaces, cafes, restaurants, or clubs (16%). Support group meetings were most often held monthly (48%) or bi-monthly (42%).

**Table 2:** HNC support group characteristics

Characteristic	*n* (%)
Year of establishment	
Before 2000	3 (16)
2000–2020	12 (63)
After 2020	4 (21)
Region	
Regional or rural area	6 (32)
Urban area or a major city	13 (68)
Location	
Public hospital	9 (48)
Community space	3 (16)
Café, restaurant, or club	3 (16)
Office building	1 (5)
Online only	1 (5)
Other	2 (11)
Frequency	
Monthly	9 (48)
Bi-monthly	8 (42)
Quarterly	1 (5)
Non-scheduled virtual engagement	1 (5)
Topics or experiences covered^a^	
Sharing experiences	19 (100)
Care after treatment	17 (89)
Side effects of treatment	17 (89)
Wellbeing	16 (84)
Dental care	14 (74)
Guest speakers	14 (74)
Nutrition	14 (74)
Socializing	14 (74)
Treatment	13 (68)
Fear of cancer recurrence	11 (58)
Mental health	11 (58)
Fundraising	2 (11)
Others	2 (11)
Promotion methods^a^	
Word of mouth	15 (79)
Mailing list	14 (74)
Flyers	11 (58)
Hospital notice boards	8 (42)
Social media (Facebook/Instagram)	8 (42)
Website (HANCA/ANZHNCS)	3 (16)
Community notice boards	2 (11)
Health professional referral	1 (5)
Funding sources	
Self-funded by facilitator	7 (37)
Donations, public	3 (16)
In-kind contributions	3 (16)
Part of core business	3 (16)
Donations, private	2 (11)
Self-funded by group members	2 (11)
Grant funding, local	1 (5)
No fund	1 (5)

^a^Multiple responses possible

*HANCA*, Head and Neck Cancer Australia

*ANZHNCS*, Australia and New Zealand Head and Neck Cancer Society

A large variety of topics were discussed at support groups. All groups reported that sharing experiences was a topic, with other frequently covered topics including care after treatment (89%), side effects of treatment (89%), and wellbeing (84%) (Table 2). Word of mouth (79%) and mailing lists (74%) were the primary promotion methods, and the predominant funding source was self-funding by facilitators (37%).

Based on the questionnaire results, the primary challenges faced by participants running HNC support groups were identified and ranked (Table 3). The top three challenges were administration, content, and location.

**Table 3:** Priority challenges for participants running a HNC support group

Ranking	Challenge area	Total points
1	Administration	31
2	Content	17
3	Location	12
4	Funding	9
5	Staffing	9
6	Recruitment	7
7	Scheduling	6
8	Ongoing training	5

### Support group guidelines and training

About one-third (32%) of participants reported receiving guidelines when they first facilitated HNC support group, and all participants who received guidelines found them useful (Table 4). Of the 13 participants who reported they did not receive guidelines, most (92%) thought that guidelines would have been useful. Furthermore, 42% of the participants underwent support group facilitation training, and unanimously (100%) deemed the training beneficial.

**Table 4:** HNC support group guideline and training characteristics

Characteristic	Participants *n* (%)
Received guidelines at first participation	
Yes	6 (32)
* Cancer council training and guidelines*	*5 (83)*
* Western & Central Melbourne Integrated Cancer Service*	*1 (17)*
* Internal or departmental training*	*1 (17)*
Effectiveness of guidelines	
Useful (of those who *had* received guidelines)	6 (100)
Perceived usefulness of guidelines	
Useful (of those who *had not* received guidelines)	12 (92)
Received training for establishing or running a support group	
Yes	8 (42)
* Cancer council training*	*4 (50)*
* Western & Central Melbourne Integrated Cancer Service Training*	*2 (25)*
* Professional development opportunities & supervision provided by the department*	*1 (13)*
* Site-specific training*	*1 (13)*
Effectiveness of training	
Useful (of those who received training)	8 (100)
Preferred source of training	
HANCA	12 (63)
ANZHNCS	4 (21)
Cancer council	2 (11)
A clinical team	1 (5)
Preferred medium of training	
In-person	8 (42)
Online materials	6 (32)
Printed materials	3 (16)
Video conferencing	2 (11)
Helped the most in running a support group (*n* = 16)	
Team and administrative support	6 (38)
Professional relationships and experience	5 (31)
Peer and collaborative support	4 (25)
Technology and process improvement	1 (6)

*HANCA*, Head and Neck Cancer Australia

*ANZHNCS*, Australia and New Zealand Head and Neck Cancer Society

The preferred sources for the guidelines and training among participants were primarily HANCA (63%), and 42% expressed a preference for in-person formats for the training. Sixteen of the 19 participants provided a response to a question about what factors helped most in running a support group, with team and administrative support (38%) and professional relationships and experience (31%) identified as the most helpful for running a HNC support group.

### Additional insights into HNC support groups

**Fig. 1:**
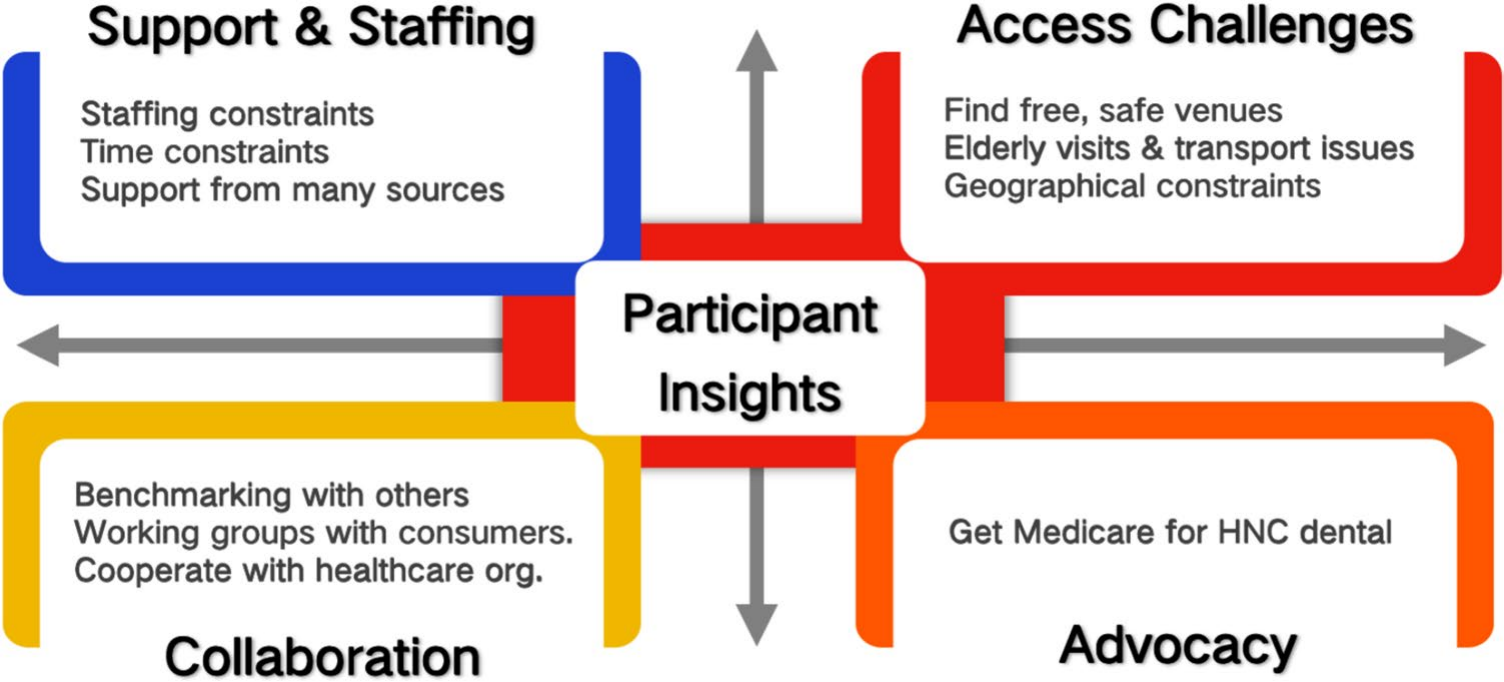
Insights into the HNC support group

Analysis of open-ended questions at the end of the survey revealed that facilitators of HNC groups were facing a number of challenges and bottlenecks in their operation (Fig. 1). Firstly, the lack of administrative support for organizers has led some groups to discontinue activities, while existing organizers often experience immense time pressures. These pressures stem from a range of responsibilities, encompassing but not limited to coordinating meetings and training sessions, managing communications with members, applying for funding or reimbursements. The cumulative burden of these tasks, often carried out on a voluntary basis and alongside full-time professional or personal commitments, makes it challenging for facilitators to sustain group activities in the long term. Secondly, practical needs such as venue availability, transportation accessibility, remote participation options, and limited geographic coverage severely hinder patient participation. Strengthening collaboration with professional bodies like hospital cancer centers and cancer associations, sharing experiences with regional organizations, and forming working groups with consumer representatives could help improve service quality. Finally, participants called on the government and relevant authorities to advocate for policy benefits such as dental coverage for patients with HNC.

## Discussion

### Summary of main findings and interpretation of results

This study utilized a survey with both quantitative and qualitative components to collect and analyze information on the characteristics, gaps, training status, attitudes, and expectations of HNC support group facilitators, founders, and collaborators in Australia.

Approximately one-third of support groups are located in rural areas, while two-thirds are in urban areas or major cities. This distribution is encouraging as it broadly reflects the geographic distribution of the Australian population, with approximately one-third of Australians living in rural areas. Nearly half of all support groups hold their activities in hospitals, and only one HNC support group operates online, which services people in remote and rural areas. The predominance of in-person meetings can pose access issues, especially given the greater geographic distances in rural areas. Compared to urban areas, HNC patients in rural areas face greater inequalities in accessing healthcare services, resulting in a higher rehabilitation burden for rural populations and an increased need for sustainable healthcare services and expertise [26]. Additionally, HNC patients in rural areas experience greater economic, emotional, and psychological stress, with a higher risk of suicide [26, 27]. Addressing the needs of HNC patients, especially those in rural areas, requires careful consideration. A conveniently located and cost-effective space positively impacts the sustainability of support groups. Additionally, the flexibility of online support groups in terms of time and location may enhance their sustainability [17], especially for those in regional or rural areas [28]. While online support groups offer potential benefits in improving accessibility, the shift toward digital formats may also give rise to unintended forms of exclusion. Individuals with limited digital literacy, inadequate access to technological devices, or unreliable internet connectivity may face substantial barriers to participation [29]. To ensure equitable access, it is essential that the development and implementation of online support services incorporate strategies to mitigate the digital divide and accommodate the diverse technological capacities and socioeconomic contexts of HNC patients [17, 29].

The content of support group activities predominantly covers sharing experiences, care after treatment, and managing side effects of treatment, effectively addressing the personalized psychological needs of HNC patients as well as providing ongoing physical care support [30]. The content covered in support group activities generally aligns well with the needs and expectations of HNC patients [21]. Notably, three-quarters of the support groups include topics on dental health, which is a major concern for most HNC patients, requiring their understanding and management [21, 31].

The majority (79%) of support groups are promoted through word of mouth, with only one group using referral by health professionals. Given the frequent interactions between health professionals and patients, having health professionals promoting HNC support groups to address various patient needs could be highly beneficial. This approach not only helps meet the psychosocial support needs of HNC patients, which may not be fully addressed during consultations with health professionals [32], but also enhances the visibility of HNC support groups. Broader promotional strategies could also attract more funding, addressing one of the key limitations to the sustainability of HNC support groups.

A large proportion of support groups are self-funded or rely on donations, including in-kind contributions, and only one support group received local grant funding. Funds are typically used for expenses such as food, coffee, or tea, renting a venue for activities, or covering fees for online platforms used by virtual support groups [17]. The relatively low financial requirements may explain why many support groups can operate on self-funding or donations. However, the limited external funding means that facilitators and collaborators often need to dedicate substantial time and effort to sustain the support group. Consequently, they may be unable to continuously donate money or time, raising concerns about the sustainability of support groups [17, 33].

Administration is identified as the biggest challenge in the operation of support groups. Typically, the responsibility of administration falls on the founders and facilitators of the support groups [17]. The lack of centralized tracking systems and limited administrative support can impede the effectiveness of these groups [17, 34]. When faced with financial, time, and administrative pressures, if the founders or facilitators decide to retire or withdraw, the support groups risk disintegration, leaving HNC patients with increased helplessness and confusion [35].

Approximately one-third of participants received guidelines and training upon initially joining the HNC support group. Currently, the primary sources of these guidelines and training are the Cancer Council and the Western & Central Melbourne Integrated Cancer Service [36, 37]. Both were considered useful by participants and are freely available resources. The Cancer Council, being a non-profit organization, provides extensive support and services to various cancer patients and their families, but it is not specifically tailored to HNC. Notably, over four-fifths of participants reported a desire to receive guidance and training from HANCA and Australia and New Zealand Head and Neck Cancer Society (ANZHNCS). This likely relates to the specialized research these organizations conduct on HNC.

### Meaning of the study with possible implications for practice, policy, and research

This article is based on a list of 26 HNC support groups, which may not sufficiently represent groups in regional or remote areas [24]. However, considering the need for support in these areas, more online groups could improve accessibility. Collaboration between HNC support groups, hospitals, and cancer associations can enhance service quality and visibility, encouraging more medical professionals to recommend these groups. Most HNC support groups rely on self-funding or donations, facing long-term sustainability challenges. Partnering with relevant institutions to seek government funding and patient benefits is crucial. Professional HNC-focused organizations, like HANCA and ANZHNCS, may also be able to address the current lack of relevant guidelines and training for HNC support groups. The establishment of a guidance and training system could be part of their future work. Future research should focus on larger, more in-depth studies to understand the needs and feedback of various stakeholders, including utilizing qualitative research methods to elicit richer quality of data.

### Strengths and limitations

This study on HNC support groups in Australia employed a mixed-methods approach, combining quantitative and qualitative data to provide a comprehensive perspective on the current state of these groups. While the findings of this study are influenced by the Australian context—including healthcare service structures, population geography, and the fragmented nature of support group facilitation—many of the challenges identified, such as limited administrative support, uneven funding, and unequal access to services in remote areas, are common to other high-income settings [38]. Therefore, the strategies proposed in this study—such as providing structured training, centralizing administrative assistance, and developing convenient online or hybrid models—may have wider applicability beyond Australia.

The research focuses on group facilitators rather than patients, offering preliminary insights into the challenges and future directions of HNC support groups. However, the study has limitations, including a small sample size of 19 group facilitators, which may limit representativeness. The study did not examine the specific content of guidelines or facilitator training, which limits insight into support quality. The survey did not capture data on group accessibility for individuals with severe communication impairments, limiting understanding of how well current formats accommodate diverse functional needs. The questionnaire content primarily focused on the structure and format of the HNC support group and the role of the facilitator, so no specific information at the patient level was obtained, which may limit understanding of patients’ direct experiences. Furthermore, relying solely on a questionnaire for data collection may have constrained the depth of qualitative insights, as the format did not permit follow-up questions or more detailed exploration of participants’ views.

## Conclusions

This study on HNC support groups in Australia offers a comprehensive perspective on the current state of these groups. While the research provides valuable insights into the challenges and operational aspects from the facilitators’ viewpoint, it highlights the need for improved administrative support and sustainable funding. The findings suggest that collaboration with professional bodies and the establishment of online support groups could enhance accessibility and service quality. Future research should focus on larger-scale studies to incorporate patient perspectives and address the multifaceted needs of HNC patients more effectively.

## Supplementary Information

Below is the link to the electronic supplementary material.

Supplementary file1 (PDF 217 KB)

## Data Availability

The data that support the findings of this study are available from the authors upon reasonable request and with approval from an appropriately constituted Human Research Ethics Committee.
