# Supplementary material for: Challenges and opportunities: a mixed-methods study on the current status of head and neck cancer support groups in Australia
Source: Support Care Cancer. 2025 Jul 11;33(8):684. doi: 10.1007/s00520-025-09752-8 (PMC12254088; doi:10.1007/s00520-025-09752-8)
Supplement: Supplementary file 1 — Supplementary file1 (PDF 217 KB) [file 520_2025_9752_MOESM1_ESM.pdf]

Guidelines and models of delivery in head and neck cancer support groups

Q. I am a Facilitator, Founder or Co-ordinator of a Head and Neck Cancer Support Group:

[branching logic employed]

- ☐ Yes
- ☐ No

  If no, Thank you for your interest in our study, unfortunately you are not eligible to participate.

PART A: E-Consent

Participant Information Sheet

[If Yes]

This Participant Information Sheet (PIS) will tell you what is involved in the study and help you decide whether you wish to take part. Please read this information carefully.

If there is anything you do not understand or if you feel you need more information about anything, please ask.

Before you decide, please feel free to talk things over with a colleague or friend.

[Attachment: PIS]

Study Procedures

If you agree to participate in this study, you will be asked to complete an online questionnaire. In this questionnaire, comprising tick boxes and free-text responses, you will be asked to share information about yourself and details about your Head and Neck Cancer support group.

If the study data will be used for future research purposes and / or shared with national and international collaborators, Ethics Approval will be required to be sought prior to access any non-identifiable data.

Online Consent

Q. Have you read and understood the Participant Information Sheet?

- ☐ Yes
- ☐ No

  If no, you are required to read the PIS before agreeing to participate in this study. Please take the time to download the PIS before proceeding.

Q. Statement

- I have been made aware of the procedures involved in the study, including any known or expected inconvenience, risk, discomfort or potential side effect and of their implications as far as they are currently known by the researchers.
- I understand that my de-identified data may be used for future research, and I agree to this.
- I have had an opportunity to ask questions and I am satisfied with the answers I have received. I freely choose to participate in this study and understand that I can withdraw at any time.
- I also understand that the research study is strictly confidential.

Q. I hereby agree to participate in this research study:

- ☐ Yes
- ☐ No

  If no, Thank you for your interest in our study.

Q5. I consent to the storage and use of my information collected from me for use, as described (tick all that apply):

- ☐ This specific research project;
- ☐ Other research that is closely related to this relevant research project.

PART B: Questionnaire

Section 1: A Little About You

Q. Please tell us your identified gender:

- ☐ Female
- ☐ Male
- ☐ Other__________________

Q. Please tell us your age:

- ☐ 18 – 29
- ☐ 30 – 45
- ☐ 46 – 59
- ☐ 60 – 75
- ☐ 76 years and over

Q. Please tell us your profession or role as facilitator, founder or co-ordinator (select all that apply)

- ☐ Founder
- ☐ Facilitator
- ☐ Co-ordinator
- ☐ Dietitian
- ☐ Nurse
- ☐ Physiotherapist
- ☐ Speech Pathologist
- ☐ Patient Advocate
- ☐ Other_________________________

Q. What best describes your current employment status?

- ☐ Full-time
- ☐ Part-time
- ☐ Casual
- ☐ Home duties
- ☐ Contract
- ☐ Freelance
- ☐ Unemployed
- ☐ Retired

Q. Have you previously received a Head and Neck Cancer diagnosis:

- ☐ Yes
- ☐ No

Q. Have you previously cared for someone with a Head and Neck Cancer diagnosis:

- ☐ Yes
- ☐ No

Q. Do you currently hold any qualifications relevant to support group facilitation:

- ☐ Yes, please specify_______________
- ☐ No
- ☐ I'm not sure

Section 2: A Little About Your Support Group

Q. What year was your support group established?

- ☐ Please specify__________________________

Q. What <u>best</u> describes the setting of where your support group takes place?

- ☐ Hospital, Private
- ☐ Hospital, Public
- ☐ Office building
- ☐ Community space
- ☐ Café, Restaurant or Club
- ☐ Online only
- ☐ Other__________________________

Q. What <u>best</u> describes the area where your support group takes place?

- ☐ Urban area or a major city
- ☐ Regional or rural area
- ☐ Not applicable, online only

Q. What <u>best</u> describes the frequency of your support group?

- ☐ Weekly
- ☐ Fortnightly
- ☐ Monthly
- ☐ Bi-monthly
- ☐ Quarterly

Q. What <u>topics or experiences</u> does your support group cover? Tick all that apply

- ☐ Treatment
- ☐ Side effects of treatment
- ☐ Care after treatment
- ☐ Nutrition
- ☐ Dental Care
- ☐ Fear of cancer recurrence
- ☐ Wellbeing
- ☐ Mental health
- ☐ Sharing experiences
- ☐ Socialising
- ☐ Guest speakers
- ☐ Fundraising

Q. How do you promote your support group? Tick all that apply

- ☐ Mailing list

- ☐ Notice boards, Community
- ☐ Notice boards, Hospital
- ☐ Word of mouth
- ☐ Flyers
- ☐ Facebook
- ☐ Twitter/X
- ☐ Instagram
- ☐ TikTok
- ☐ Snapchat
- ☐ WhatsApp
- ☐ LinkedIn
- ☐ Other, please specify_______________

Q. How is your support group funded? Tick all that apply

- ☐ Grant funding, local
- ☐ Grant funding, state
- ☐ Donations, public
- ☐ Donations, private
- ☐ Self-funded by facilitator
- ☐ Self-funded by group members
- ☐ Other____________________

Section 3: A Little About Your Support Group Training

Q. When you first became involved, were you aware of any guidelines to help you?

- ☐ Yes, please specify_______________
- ☐ No

Q. Were guidelines useful? / Would guidelines have been useful?

[branching logic employed from above question]

- ☐ Yes
- ☐ No

Q. Why/why not?

[free text response]

Q. Did you receive any training in establishing or running a support group?

- ☐ Yes, please specify_______________
- ☐ No

Q. Was training useful? / Would training have been useful?

[branching logic employed from above question]

- ☐ Yes
- ☐ No

Q. Why/why not?

[free text response]

Q. If guidelines and/or training were available, who would you prefer to receive it from?

- ☐ Australia and New Zealand Head and Neck Cancer Society (ANZHNCS)
- ☐ Cancer Council
- ☐ Head and Neck Cancer Australia (HANCA)
- ☐ A clinical team
- ☐ Another support group

Q. If guidelines and/or training were available, how would you prefer to receive them?

- ☐ In-person
- ☐ Video conferencing
- ☐ Online materials
- ☐ Printed materials

Q. In your experience, what are the 3 biggest challenges in running a support group?

- ☐ Administration
- ☐ Funding
- ☐ Staffing
- ☐ Recruitment
- ☐ Scheduling
- ☐ Content
- ☐ Location
- ☐ Training, ongoing
- ☐ Other, please describe below

[free text response]

Q. In your experience, what has helped you the most in running a support group?

[free text response]

Q. Is there anything else you would like to say on this topic before we finish?

[free text response]

Please press SUBMIT below when you have completed the questionnaire.

Thank you! We are grateful for your participation in our research study.
